# [Disulfanediylbis(ferrocenyl­thio­phosphinato)-κ^2^ *O*,*O*]titanocene tetra­hydro­furan tris­olvate

**DOI:** 10.1107/S1600536812010811

**Published:** 2012-03-17

**Authors:** Mehmet Karakus, J. Derek Woollins, Peter Lönnecke, Evamarie Hey-Hawkins

**Affiliations:** a Department of Chemistry, Faculty of Arts and Sciences, Pamukkale University, Kinikli, 20017 Denizli, Turkey; b School of Chemistry, University of St Andrews, St Andrews, Fife KMY16 9ST, Scotland; c Institut für Anorganische Chemie, Universität Leipzig, Johannisallee 29, D-04103 Leipzig, Germany

## Abstract

The title compound, [Fe_2_Ti(C_5_H_5_)_4_)(C_10_H_8_O_2_P_2_S_4_)]·3C_4_H_8_O, contains a central seven-membered TiO_2_P_2_S_2_ ring with a very similar geometry compared to the derivative showing anisyl instead of ferrocenyl substituents, the Ti—O distance being marginally longer for the anisyl derivative. Two tetra­hydro­furan solvent mol­ecules are each disordered on a twofold axis.

## Related literature

For a closely related analogue with anisyl rather than ferrocenyl groups, see: Zank & Rauchfuss (1985 ▶). For other cyclo­penta­dienyl titanium phosphous sulfides, see: Zank & Rauchfuss (1984 ▶).
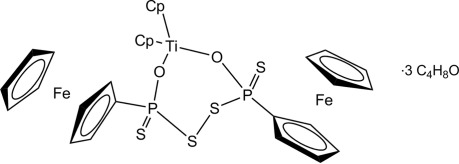


## Experimental

### 

#### Crystal data

[Fe_2_Ti(C_5_H_5_)_4_(C_10_H_8_O_2_P_2_S_4_)]·3C_4_H_8_O
*M* *_r_* = 986.62
Orthorhombic, 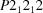

*a* = 15.9784 (3) Å
*b* = 30.6984 (8) Å
*c* = 8.7072 (2) Å
*V* = 4270.98 (17) Å^3^
*Z* = 4
Mo *K*α radiation
μ = 1.17 mm^−1^
*T* = 130 K
0.35 × 0.2 × 0.03 mm

#### Data collection

Oxford Diffraction Xcalibur Sapphire3 Gemini diffractometer
Absorption correction: multi-scan (*CrysAlis PRO*; Oxford Diffraction, 2009 ▶) *T*_min_ = 0.944, *T*_max_ = 1
48502 measured reflections
8729 independent reflections
7146 reflections with *I* > 2σ(*I*)
*R*_int_ = 0.056

#### Refinement

*R*[*F* ^2^ > 2σ(*F* ^2^)] = 0.048
*wR*(*F* ^2^) = 0.105
*S* = 1.01
8729 reflections
481 parameters
70 restraints
H-atom parameters constrained
Δρ_max_ = 0.66 e Å^−3^
Δρ_min_ = −0.65 e Å^−3^
Absolute structure: Flack (1983 ▶), 3819 Friedel pairs
Flack parameter: 0.122 (19)

### 

Data collection: *CrysAlis PRO* (Oxford Diffraction, 2009 ▶); cell refinement: *CrysAlis PRO*; data reduction: *CrysAlis PRO*; program(s) used to solve structure: *SHELXS97* (Sheldrick, 2008 ▶); program(s) used to refine structure: *SHELXL97* (Sheldrick, 2008 ▶); molecular graphics: *DIAMOND* (Brandenburg, 2005 ▶); software used to prepare material for publication: *WinGX* (Farrugia, 1999 ▶).

## Supplementary Material

Crystal structure: contains datablock(s) I, global. DOI: 10.1107/S1600536812010811/im2362sup1.cif

Structure factors: contains datablock(s) I. DOI: 10.1107/S1600536812010811/im2362Isup2.hkl

Additional supplementary materials:  crystallographic information; 3D view; checkCIF report

## Figures and Tables

**Fig. 1.**
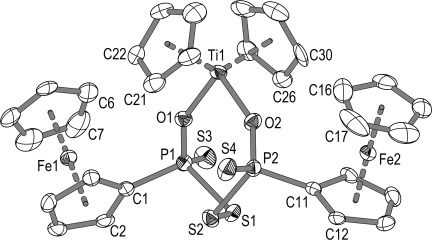
Molecular structure of (1) with displacement ellipsoids drawn at the 50% probability level, hydrogen atoms are omitted for clarity

## supplementary crystallographic information

### Comment

Mixed metal heterobimetallic complexes are of intrest since they contain an electron-defficient early transition metal and an electron-rich late transition metal in one complex. This paper descibes a rare example of a titanium/iron system.

### Experimental

[*t*BuNH_3_][Fe(C_5_H_5_){C_5_H_4_P(O-Bornyl)S_2_}] (1) (0.50 g, 0.985 mmol) was added to a solution of [Cp_2_TiCl_2_] (0.123 g, 0.492 mmol) in THF (20 ml). After 30 minutes *t*BuNH_3_Cl precipitated from the dark red solution. The reaction mixture was filtered and the solution was cooled to -18 °C giving red crystals which were isolated and dried *in vacuo*. Yield: 0.28 g (74%), m.p.: 176 °C (decomp). Anal. calc. for C_30_H_20_O_2_P_2_S_4_Fe_2_Ti (%): C 46.77; H 3.66; Found: C 47.18; H 3.85.

### Refinement

The structure was refined as an racemic twin (ratio is 0.78 (2):0.12 (2)). All non-hydrogen atoms were refined anisotropically except those in disordered thf solvent molecules. All H atoms were calculated in idealized positions. With the exception of one thf molecule (O(3), C(31) - C(34)), all other solvent molecules are disordered, two of them (O(5), O(6), C(39) - C(46)) on a C2-axis. All thf solvent molecules are non-coordinating and located on (101)-layers.

### Crystal data

**Table:**

[Fe_2_Ti(C_5_H_5_)_4_(C_10_H_8_O_2_P_2_S_4_)]·3C_4_H_8_O	*F*(000) = 2048
*M**_r_* = 986.62	*D*_x_ = 1.534 Mg m^−^^3^
Orthorhombic, *P*2_1_2_1_2	Mo *K*α radiation, λ = 0.71073 Å
Hall symbol: P 2 2ab	Cell parameters from 16673 reflections
*a* = 15.9784 (3) Å	θ = 2.9–28.0°
*b* = 30.6984 (8) Å	µ = 1.17 mm^−^^1^
*c* = 8.7072 (2) Å	*T* = 130 K
*V* = 4270.98 (17) Å^3^	Flattened needle, red
*Z* = 4	0.35 × 0.2 × 0.03 mm

### Data collection

**Table:**

Oxford Diffraction Xcalibur Sapphire3 Gemini diffractometer	8729 independent reflections
Graphite monochromator	7146 reflections with *I* > 2σ(*I*)
Detector resolution: 16.356 pixels mm^-1^	*R*_int_ = 0.056
ω scans	θ_max_ = 26.4°, θ_min_ = 2.9°
Absorption correction: multi-scan (*CrysAlis PRO*; Oxford Diffraction, 2009)	*h* = −19→19
*T*_min_ = 0.944, *T*_max_ = 1	*k* = −38→38
48502 measured reflections	*l* = −10→10

### Refinement

**Table:**

Refinement on *F*^2^	Secondary atom site location: difference Fourier map
Least-squares matrix: full	Hydrogen site location: inferred from neighbouring sites
*R*[*F*^2^ > 2σ(*F*^2^)] = 0.048	H-atom parameters constrained
*wR*(*F*^2^) = 0.105	*w* = 1/[σ^2^(*F*_o_^2^) + (0.058*P*)^2^] where *P* = (*F*_o_^2^ + 2*F*_c_^2^)/3
*S* = 1.01	(Δ/σ)_max_ = 0.001
8729 reflections	Δρ_max_ = 0.66 e Å^−^^3^
481 parameters	Δρ_min_ = −0.65 e Å^−^^3^
70 restraints	Absolute structure: Flack (1983), 3819 Friedel pairs
Primary atom site location: structure-invariant direct methods	Flack parameter: 0.122 (19)

### Special details

**Table:**

Geometry. All s.u.'s (except the s.u. in the dihedral angle between two l.s. planes) are estimated using the full covariance matrix. The cell s.u.'s are taken into account individually in the estimation of s.u.'s in distances, angles and torsion angles; correlations between s.u.'s in cell parameters are only used when they are defined by crystal symmetry. An approximate (isotropic) treatment of cell s.u.'s is used for estimating s.u.'s involving l.s. planes.
Refinement. Refinement of *F*^2^ against ALL reflections. The weighted *R*-factor *wR* and goodness of fit *S* are based on *F*^2^, conventional *R*-factors *R* are based on *F*, with *F* set to zero for negative *F*^2^. The threshold expression of *F*^2^ > 2σ(*F*^2^) is used only for calculating *R*-factors(gt) *etc*. and is not relevant to the choice of reflections for refinement. *R*-factors based on *F*^2^ are statistically about twice as large as those based on *F*, and *R*- factors based on ALL data will be even larger.

### Fractional atomic coordinates and isotropic or equivalent isotropic displacement parameters (Å^2^)

**Table:**

	*x*	*y*	*z*	*U*_iso_*/*U*_eq_	Occ. (<1)
Fe1	0.89358 (4)	0.15429 (2)	0.34717 (8)	0.02543 (17)	
Fe2	0.77379 (4)	0.37979 (2)	1.13086 (7)	0.02432 (16)	
Ti1	0.97367 (5)	0.28801 (3)	0.74545 (10)	0.02154 (19)	
S1	0.75175 (7)	0.22155 (4)	0.81125 (14)	0.0289 (3)	
S2	0.71563 (7)	0.27539 (4)	0.68638 (14)	0.0292 (3)	
S3	0.89057 (9)	0.14787 (4)	0.80578 (15)	0.0364 (3)	
S4	0.75252 (8)	0.37627 (4)	0.66252 (14)	0.0343 (3)	
P1	0.85645 (7)	0.19835 (4)	0.68687 (14)	0.0220 (3)	
P2	0.77803 (7)	0.32774 (4)	0.79565 (13)	0.0223 (3)	
O1	0.91691 (18)	0.23650 (10)	0.6634 (4)	0.0229 (7)	
O2	0.86968 (18)	0.31296 (10)	0.8211 (4)	0.0253 (7)	
C1	0.8189 (3)	0.18549 (15)	0.4993 (5)	0.0202 (10)	
C2	0.7829 (3)	0.14437 (16)	0.4575 (6)	0.0264 (11)	
H2	0.7701	0.1209	0.5246	0.032*	
C3	0.7701 (3)	0.14566 (17)	0.2963 (6)	0.0314 (12)	
H3	0.7469	0.1227	0.2366	0.038*	
C4	0.7970 (3)	0.18604 (17)	0.2388 (6)	0.0317 (12)	
H4	0.795	0.1951	0.1346	0.038*	
C5	0.8276 (3)	0.21076 (16)	0.3638 (5)	0.0251 (10)	
H5	0.8501	0.2394	0.3581	0.03*	
C6	1.0126 (3)	0.1511 (2)	0.4333 (6)	0.0457 (16)	
H6	1.0328	0.1657	0.5222	0.055*	
C7	0.9766 (4)	0.1096 (2)	0.4295 (7)	0.0537 (18)	
H7	0.9677	0.091	0.5152	0.064*	
C8	0.9554 (4)	0.1001 (2)	0.2729 (8)	0.0519 (18)	
H8	0.9301	0.0741	0.236	0.062*	
C9	0.9789 (3)	0.1362 (2)	0.1841 (7)	0.0424 (14)	
H9	0.9724	0.1392	0.0762	0.051*	
C10	1.0139 (3)	0.1673 (2)	0.2840 (6)	0.0389 (14)	
H10	1.035	0.195	0.2543	0.047*	
C11	0.7355 (3)	0.33281 (15)	0.9827 (5)	0.0210 (10)	
C12	0.6671 (3)	0.35989 (16)	1.0269 (5)	0.0225 (11)	
H12	0.6338	0.3768	0.9591	0.027*	
C13	0.6570 (3)	0.35751 (16)	1.1883 (6)	0.0292 (12)	
H13	0.616	0.3725	1.247	0.035*	
C14	0.7193 (3)	0.32859 (15)	1.2473 (6)	0.0306 (11)	
H14	0.7267	0.3208	1.352	0.037*	
C15	0.7677 (3)	0.31369 (14)	1.1234 (5)	0.0234 (10)	
H15	0.8139	0.2943	1.1305	0.028*	
C16	0.8714 (5)	0.4125 (3)	1.0369 (7)	0.065 (2)	
H16	0.9014	0.4044	0.9471	0.078*	
C17	0.7993 (5)	0.4391 (2)	1.0412 (8)	0.064 (2)	
H17	0.7717	0.4521	0.9562	0.077*	
C18	0.7766 (4)	0.44247 (17)	1.1973 (9)	0.0529 (17)	
H18	0.7302	0.4585	1.2357	0.063*	
C19	0.8327 (3)	0.41875 (17)	1.2864 (7)	0.0382 (14)	
H19	0.831	0.4158	1.395	0.046*	
C20	0.8914 (3)	0.40014 (18)	1.1894 (7)	0.0419 (14)	
H20	0.937	0.3822	1.2197	0.05*	
C21	0.9416 (3)	0.31887 (18)	0.5019 (6)	0.0334 (13)	
H21	0.8864	0.3159	0.4627	0.04*	
C22	1.0082 (3)	0.28970 (18)	0.4770 (5)	0.0290 (12)	
H22	1.0067	0.264	0.4164	0.035*	
C23	1.0781 (3)	0.30601 (18)	0.5600 (6)	0.0353 (13)	
H23	1.132	0.293	0.5654	0.042*	
C24	1.0533 (3)	0.34478 (18)	0.6323 (7)	0.0387 (13)	
H24	1.0874	0.3624	0.6965	0.046*	
C25	0.9706 (3)	0.35283 (16)	0.5938 (6)	0.0308 (11)	
H25	0.9388	0.3775	0.625	0.037*	
C26	0.9764 (3)	0.25151 (18)	0.9892 (6)	0.0294 (11)	
H26	0.9279	0.237	1.0258	0.035*	
C27	1.0395 (3)	0.23318 (17)	0.8989 (6)	0.0320 (12)	
H27	1.0415	0.2038	0.8643	0.038*	
C28	1.0988 (3)	0.26523 (18)	0.8682 (7)	0.0407 (14)	
H28	1.1483	0.2615	0.8094	0.049*	
C29	1.0727 (4)	0.3041 (2)	0.9391 (7)	0.0419 (15)	
H29	1.101	0.3312	0.9353	0.05*	
C30	0.9975 (4)	0.2955 (2)	1.0165 (6)	0.0381 (14)	
H30	0.9664	0.3157	1.0764	0.046*	
O3	0.3263 (4)	0.49316 (18)	0.7990 (7)	0.0938 (18)	
C31	0.2642 (6)	0.4823 (4)	0.6876 (13)	0.131 (4)	
H31A	0.246	0.4517	0.701	0.157*	
H31B	0.2147	0.5014	0.6993	0.157*	
C32	0.3004 (6)	0.4880 (3)	0.5409 (11)	0.099 (3)	
H32A	0.2579	0.4986	0.4671	0.119*	
H32B	0.3232	0.4601	0.5027	0.119*	
C33	0.3724 (6)	0.5224 (3)	0.5582 (11)	0.089 (3)	
H33A	0.4251	0.5121	0.511	0.106*	
H33B	0.3567	0.5508	0.5126	0.106*	
C34	0.3790 (4)	0.5250 (2)	0.7236 (10)	0.070 (2)	
H34A	0.3627	0.5546	0.7577	0.084*	
H34B	0.4379	0.5202	0.7542	0.084*	
O4	0.6390 (4)	0.0869 (2)	0.0514 (8)	0.0610 (15)*	0.705 (9)
C35	0.5894 (6)	0.0503 (3)	0.0012 (11)	0.0610 (15)*	0.705 (9)
H35A	0.6089	0.0238	0.055	0.073*	0.705 (9)
H35B	0.5303	0.0552	0.0308	0.073*	0.705 (9)
C36	0.5932 (6)	0.0431 (3)	−0.1565 (10)	0.0610 (15)*	0.705 (9)
H36A	0.5389	0.0495	−0.2063	0.073*	0.705 (9)
H36B	0.6096	0.0127	−0.1797	0.073*	0.705 (9)
C37	0.6642 (6)	0.0769 (3)	−0.2110 (11)	0.0610 (15)*	0.705 (9)
H37A	0.7067	0.0623	−0.2757	0.073*	0.705 (9)
H37B	0.6394	0.1014	−0.2695	0.073*	0.705 (9)
C38	0.7005 (6)	0.0921 (3)	−0.0681 (11)	0.0610 (15)*	0.705 (9)
H38A	0.7512	0.0749	−0.0434	0.073*	0.705 (9)
H38B	0.7167	0.1231	−0.0773	0.073*	0.705 (9)
O4F	0.6213 (13)	0.0919 (8)	−0.004 (2)	0.102 (5)*	0.295 (9)
C35F	0.7051 (15)	0.0729 (11)	−0.013 (3)	0.102 (5)*	0.295 (9)
H35C	0.7475	0.0944	0.0208	0.122*	0.295 (9)
H35D	0.7088	0.0471	0.0554	0.122*	0.295 (9)
C36F	0.7205 (14)	0.0603 (10)	−0.168 (3)	0.102 (5)*	0.295 (9)
H36C	0.7396	0.0297	−0.1733	0.122*	0.295 (9)
H36D	0.7638	0.0792	−0.2149	0.122*	0.295 (9)
C37F	0.6340 (17)	0.0660 (10)	−0.254 (3)	0.102 (5)*	0.295 (9)
H37C	0.6407	0.0722	−0.3651	0.122*	0.295 (9)
H37D	0.5959	0.0409	−0.239	0.122*	0.295 (9)
C38F	0.6097 (16)	0.1042 (8)	−0.166 (3)	0.102 (5)*	0.295 (9)
H38C	0.5505	0.1119	−0.1859	0.122*	0.295 (9)
H38D	0.6454	0.1294	−0.1923	0.122*	0.295 (9)
O5	0.5387 (8)	0.4669 (4)	0.1620 (15)	0.089 (2)*	0.5
C39	0.4455 (10)	0.4703 (5)	0.153 (3)	0.089 (2)*	0.5
H39A	0.4272	0.4605	0.0497	0.106*	0.5
H39B	0.4205	0.4504	0.2294	0.106*	0.5
C40	0.4158 (10)	0.5113 (6)	0.178 (2)	0.089 (2)*	0.5
H40A	0.4056	0.5165	0.2882	0.106*	0.5
H40B	0.3633	0.5164	0.1201	0.106*	0.5
C41	0.4877 (11)	0.5410 (5)	0.116 (2)	0.089 (2)*	0.5
H41A	0.4793	0.5482	0.0067	0.106*	0.5
H41B	0.4912	0.5684	0.1762	0.106*	0.5
C42	0.5630 (10)	0.5145 (5)	0.138 (3)	0.089 (2)*	0.5
H42A	0.5946	0.5252	0.2282	0.106*	0.5
H42B	0.5995	0.5171	0.0465	0.106*	0.5
O6	−0.0552 (12)	0.4762 (6)	0.644 (2)	0.157 (4)*	0.5
C43	0.0176 (16)	0.4671 (7)	0.552 (3)	0.157 (4)*	0.5
H43A	0.0571	0.4481	0.6086	0.189*	0.5
H43B	0.0014	0.4524	0.4553	0.189*	0.5
C44	0.0543 (15)	0.5080 (8)	0.522 (3)	0.157 (4)*	0.5
H44A	0.0373	0.5191	0.4196	0.189*	0.5
H44B	0.1162	0.5063	0.526	0.189*	0.5
C45	0.0172 (17)	0.5393 (8)	0.662 (3)	0.157 (4)*	0.5
H45A	0.0301	0.529	0.7666	0.189*	0.5
H45B	0.0291	0.5708	0.6494	0.189*	0.5
C46	−0.0613 (15)	0.5260 (7)	0.605 (3)	0.157 (4)*	0.5
H46A	−0.0667	0.5312	0.4932	0.189*	0.5
H46B	−0.1081	0.5403	0.6598	0.189*	0.5

### Atomic displacement parameters (Å^2^)

**Table:**

	*U*^11^	*U*^22^	*U*^33^	*U*^12^	*U*^13^	*U*^23^
Fe1	0.0156 (3)	0.0363 (4)	0.0244 (4)	0.0052 (3)	0.0003 (3)	−0.0048 (3)
Fe2	0.0227 (3)	0.0285 (4)	0.0218 (3)	−0.0078 (3)	−0.0005 (3)	−0.0025 (3)
Ti1	0.0148 (4)	0.0256 (5)	0.0242 (4)	0.0017 (4)	−0.0026 (3)	−0.0011 (4)
S1	0.0255 (6)	0.0369 (7)	0.0242 (6)	−0.0033 (5)	0.0080 (5)	−0.0049 (5)
S2	0.0203 (6)	0.0412 (7)	0.0260 (6)	0.0064 (6)	−0.0052 (5)	−0.0105 (6)
S3	0.0434 (7)	0.0328 (7)	0.0329 (7)	−0.0007 (7)	−0.0072 (6)	0.0094 (6)
S4	0.0353 (7)	0.0406 (7)	0.0269 (7)	0.0121 (6)	0.0020 (5)	0.0058 (6)
P1	0.0211 (6)	0.0245 (6)	0.0205 (6)	0.0000 (5)	0.0014 (5)	0.0002 (5)
P2	0.0189 (6)	0.0297 (6)	0.0185 (6)	0.0058 (5)	−0.0007 (5)	−0.0018 (5)
O1	0.0191 (16)	0.0245 (17)	0.0250 (17)	0.0011 (13)	−0.0011 (13)	−0.0006 (14)
O2	0.0214 (16)	0.0286 (18)	0.0258 (18)	0.0040 (14)	0.0031 (14)	−0.0011 (14)
C1	0.014 (2)	0.024 (3)	0.023 (2)	0.006 (2)	−0.0012 (18)	−0.005 (2)
C2	0.012 (2)	0.031 (3)	0.036 (3)	0.000 (2)	0.005 (2)	−0.008 (2)
C3	0.016 (2)	0.039 (3)	0.039 (3)	0.000 (2)	0.002 (2)	−0.019 (2)
C4	0.023 (3)	0.049 (3)	0.023 (3)	0.009 (2)	0.003 (2)	−0.007 (2)
C5	0.019 (2)	0.031 (3)	0.025 (3)	0.004 (2)	−0.001 (2)	−0.003 (2)
C6	0.020 (3)	0.083 (5)	0.034 (3)	0.015 (3)	−0.003 (2)	−0.005 (3)
C7	0.041 (4)	0.075 (5)	0.044 (4)	0.034 (4)	0.010 (3)	0.013 (3)
C8	0.035 (3)	0.044 (4)	0.077 (5)	0.014 (3)	0.015 (3)	−0.018 (3)
C9	0.021 (3)	0.070 (4)	0.036 (3)	0.015 (3)	0.009 (2)	−0.007 (3)
C10	0.011 (2)	0.068 (4)	0.038 (3)	0.004 (2)	0.005 (2)	−0.003 (3)
C11	0.014 (2)	0.026 (3)	0.022 (2)	−0.002 (2)	−0.0021 (18)	−0.0040 (19)
C12	0.012 (2)	0.030 (3)	0.025 (3)	0.001 (2)	−0.0022 (18)	−0.005 (2)
C13	0.024 (2)	0.032 (3)	0.032 (3)	−0.011 (2)	0.006 (2)	−0.004 (2)
C14	0.040 (3)	0.030 (3)	0.022 (2)	−0.015 (2)	−0.002 (2)	−0.003 (2)
C15	0.022 (2)	0.027 (2)	0.021 (2)	−0.004 (2)	−0.009 (2)	0.0052 (19)
C16	0.079 (6)	0.080 (5)	0.035 (4)	−0.060 (5)	0.015 (3)	−0.014 (3)
C17	0.090 (6)	0.048 (4)	0.055 (5)	−0.048 (4)	−0.035 (4)	0.023 (3)
C18	0.044 (3)	0.025 (3)	0.089 (5)	−0.011 (3)	−0.003 (4)	−0.012 (3)
C19	0.044 (3)	0.039 (3)	0.032 (3)	−0.021 (3)	0.002 (3)	−0.005 (3)
C20	0.026 (3)	0.046 (3)	0.054 (4)	−0.017 (3)	−0.004 (3)	−0.009 (3)
C21	0.029 (3)	0.037 (3)	0.034 (3)	0.001 (3)	0.002 (2)	0.013 (3)
C22	0.030 (3)	0.035 (3)	0.023 (3)	−0.002 (2)	0.005 (2)	−0.001 (2)
C23	0.020 (3)	0.042 (3)	0.044 (3)	−0.002 (2)	0.009 (2)	0.003 (3)
C24	0.040 (3)	0.030 (3)	0.046 (3)	−0.008 (3)	0.005 (3)	0.005 (3)
C25	0.025 (3)	0.027 (3)	0.041 (3)	0.000 (2)	0.007 (2)	0.007 (2)
C26	0.021 (2)	0.040 (3)	0.026 (3)	0.002 (2)	−0.007 (2)	0.009 (2)
C27	0.027 (3)	0.036 (3)	0.033 (3)	0.013 (2)	−0.011 (2)	0.001 (2)
C28	0.020 (3)	0.055 (4)	0.047 (3)	0.002 (3)	−0.014 (3)	0.006 (3)
C29	0.037 (3)	0.038 (3)	0.050 (4)	−0.007 (3)	−0.020 (3)	0.006 (3)
C30	0.039 (3)	0.057 (4)	0.018 (3)	0.011 (3)	−0.017 (2)	−0.012 (2)
O3	0.098 (4)	0.078 (4)	0.106 (5)	−0.009 (4)	0.014 (4)	−0.012 (4)
C31	0.089 (7)	0.164 (11)	0.140 (10)	−0.040 (8)	−0.036 (8)	0.005 (9)
C32	0.090 (7)	0.120 (9)	0.089 (7)	−0.034 (7)	−0.016 (5)	−0.003 (6)
C33	0.094 (7)	0.063 (6)	0.109 (8)	−0.004 (5)	0.014 (6)	−0.010 (5)
C34	0.050 (4)	0.045 (4)	0.116 (7)	0.001 (3)	0.005 (4)	−0.008 (4)

### Geometric parameters (Å, º)

**Table:**

Fe1—C1	2.024 (4)	C22—C23	1.422 (7)
Fe1—C5	2.034 (5)	C22—H22	0.95
Fe1—C2	2.035 (5)	C23—C24	1.404 (8)
Fe1—C7	2.038 (6)	C23—H23	0.95
Fe1—C10	2.039 (5)	C24—C25	1.386 (7)
Fe1—C3	2.039 (5)	C24—H24	0.95
Fe1—C8	2.040 (6)	C25—H25	0.95
Fe1—C9	2.045 (5)	C26—C27	1.396 (7)
Fe1—C6	2.047 (5)	C26—C30	1.412 (8)
Fe1—C4	2.054 (5)	C26—H26	0.95
Fe2—C18	2.010 (5)	C27—C28	1.392 (7)
Fe2—C17	2.024 (6)	C27—H27	0.95
Fe2—C12	2.024 (4)	C28—C29	1.405 (8)
Fe2—C16	2.029 (6)	C28—H28	0.95
Fe2—C11	2.029 (4)	C29—C30	1.402 (8)
Fe2—C15	2.032 (4)	C29—H29	0.95
Fe2—C19	2.037 (5)	C30—H30	0.95
Fe2—C20	2.045 (5)	O3—C31	1.427 (9)
Fe2—C13	2.049 (5)	O3—C34	1.449 (8)
Fe2—C14	2.063 (5)	C31—C32	1.414 (10)
Ti1—O2	1.945 (3)	C31—H31A	0.99
Ti1—O1	1.958 (3)	C31—H31B	0.99
Ti1—C29	2.365 (5)	C32—C33	1.567 (10)
Ti1—C28	2.372 (5)	C32—H32A	0.99
Ti1—C24	2.372 (5)	C32—H32B	0.99
Ti1—C21	2.379 (5)	C33—C34	1.447 (10)
Ti1—C23	2.388 (5)	C33—H33A	0.99
Ti1—C25	2.389 (5)	C33—H33B	0.99
Ti1—C27	2.392 (5)	C34—H34A	0.99
Ti1—C26	2.400 (5)	C34—H34B	0.99
Ti1—C30	2.401 (5)	O4—C38	1.440 (10)
Ti1—C22	2.402 (5)	O4—C35	1.444 (10)
S1—S2	2.0607 (18)	C35—C36	1.391 (11)
S1—P1	2.1164 (16)	C35—H35A	0.99
S2—P2	2.1170 (17)	C35—H35B	0.99
S3—P1	1.9418 (17)	C36—C37	1.609 (11)
S4—P2	1.9312 (17)	C36—H36A	0.99
P1—O1	1.532 (3)	C36—H36B	0.99
P1—C1	1.784 (5)	C37—C38	1.449 (11)
P2—O2	1.549 (3)	C37—H37A	0.99
P2—C11	1.772 (5)	C37—H37B	0.99
C1—C5	1.419 (7)	C38—H38A	0.99
C1—C2	1.434 (7)	C38—H38B	0.99
C2—C3	1.419 (7)	O4F—C35F	1.464 (13)
C2—H2	0.95	O4F—C38F	1.469 (12)
C3—C4	1.404 (7)	C35F—C36F	1.428 (14)
C3—H3	0.95	C35F—H35C	0.99
C4—C5	1.414 (7)	C35F—H35D	0.99
C4—H4	0.95	C36F—C37F	1.582 (14)
C5—H5	0.95	C36F—H36C	0.99
C6—C10	1.392 (8)	C36F—H36D	0.99
C6—C7	1.398 (9)	C37F—C38F	1.458 (14)
C6—H6	0.95	C37F—H37C	0.99
C7—C8	1.435 (9)	C37F—H37D	0.99
C7—H7	0.95	C38F—H38C	0.99
C8—C9	1.403 (8)	C38F—H38D	0.99
C8—H8	0.95	O5—C39	1.496 (14)
C9—C10	1.407 (8)	O5—C42	1.527 (14)
C9—H9	0.95	C39—C40	1.362 (14)
C10—H10	0.95	C39—H39A	0.99
C11—C12	1.426 (6)	C39—H39B	0.99
C11—C15	1.453 (6)	C40—C41	1.560 (15)
C12—C13	1.416 (7)	C40—H40A	0.99
C12—H12	0.95	C40—H40B	0.99
C13—C14	1.430 (7)	C41—C42	1.464 (15)
C13—H13	0.95	C41—H41A	0.99
C14—C15	1.404 (7)	C41—H41B	0.99
C14—H14	0.95	C42—H42A	0.99
C15—H15	0.95	C42—H42B	0.99
C16—C17	1.413 (10)	O6—C43	1.439 (16)
C16—C20	1.418 (9)	O6—C46	1.571 (16)
C16—H16	0.95	C43—C44	1.412 (17)
C17—C18	1.410 (10)	C43—H43A	0.99
C17—H17	0.95	C43—H43B	0.99
C18—C19	1.391 (8)	C44—C45	1.661 (17)
C18—H18	0.95	C44—H44A	0.99
C19—C20	1.386 (8)	C44—H44B	0.99
C19—H19	0.95	C45—C46	1.407 (17)
C20—H20	0.95	C45—H45A	0.99
C21—C25	1.394 (7)	C45—H45B	0.99
C21—C22	1.407 (7)	C46—H46A	0.99
C21—H21	0.95	C46—H46B	0.99
			
C1—Fe1—C5	40.92 (19)	C13—C12—Fe2	70.6 (3)
C1—Fe1—C2	41.38 (18)	C11—C12—Fe2	69.6 (2)
C5—Fe1—C2	69.12 (19)	C13—C12—H12	125.5
C1—Fe1—C7	118.2 (2)	C11—C12—H12	125.5
C5—Fe1—C7	152.3 (2)	Fe2—C12—H12	125.8
C2—Fe1—C7	107.4 (2)	C12—C13—C14	108.0 (4)
C1—Fe1—C10	129.7 (2)	C12—C13—Fe2	68.7 (3)
C5—Fe1—C10	109.9 (2)	C14—C13—Fe2	70.2 (3)
C2—Fe1—C10	167.3 (2)	C12—C13—H13	126
C7—Fe1—C10	67.3 (3)	C14—C13—H13	126
C1—Fe1—C3	68.49 (18)	Fe2—C13—H13	126.7
C5—Fe1—C3	67.97 (19)	C15—C14—C13	108.0 (4)
C2—Fe1—C3	40.8 (2)	C15—C14—Fe2	68.8 (3)
C7—Fe1—C3	128.2 (3)	C13—C14—Fe2	69.1 (3)
C10—Fe1—C3	151.5 (2)	C15—C14—H14	126
C1—Fe1—C8	151.5 (2)	C13—C14—H14	126
C5—Fe1—C8	165.6 (2)	Fe2—C14—H14	127.7
C2—Fe1—C8	116.6 (2)	C14—C15—C11	108.7 (4)
C7—Fe1—C8	41.2 (3)	C14—C15—Fe2	71.1 (3)
C10—Fe1—C8	67.6 (3)	C11—C15—Fe2	68.9 (3)
C3—Fe1—C8	107.1 (2)	C14—C15—H15	125.6
C1—Fe1—C9	167.4 (2)	C11—C15—H15	125.6
C5—Fe1—C9	128.8 (2)	Fe2—C15—H15	125.9
C2—Fe1—C9	150.0 (2)	C17—C16—C20	108.3 (6)
C7—Fe1—C9	68.1 (2)	C17—C16—Fe2	69.4 (4)
C10—Fe1—C9	40.3 (2)	C20—C16—Fe2	70.3 (3)
C3—Fe1—C9	117.3 (2)	C17—C16—H16	125.9
C8—Fe1—C9	40.2 (2)	C20—C16—H16	125.9
C1—Fe1—C6	109.3 (2)	Fe2—C16—H16	126.1
C5—Fe1—C6	119.8 (2)	C18—C17—C16	106.1 (6)
C2—Fe1—C6	128.9 (2)	C18—C17—Fe2	69.0 (3)
C7—Fe1—C6	40.0 (3)	C16—C17—Fe2	69.8 (4)
C10—Fe1—C6	39.8 (2)	C18—C17—H17	127
C3—Fe1—C6	166.5 (3)	C16—C17—H17	127
C8—Fe1—C6	68.1 (3)	Fe2—C17—H17	125.8
C9—Fe1—C6	67.8 (2)	C19—C18—C17	109.5 (6)
C1—Fe1—C4	68.50 (19)	C19—C18—Fe2	71.0 (3)
C5—Fe1—C4	40.47 (18)	C17—C18—Fe2	70.1 (4)
C2—Fe1—C4	68.6 (2)	C19—C18—H18	125.2
C7—Fe1—C4	165.8 (3)	C17—C18—H18	125.2
C10—Fe1—C4	119.4 (2)	Fe2—C18—H18	125.3
C3—Fe1—C4	40.1 (2)	C20—C19—C18	108.2 (5)
C8—Fe1—C4	127.2 (2)	C20—C19—Fe2	70.5 (3)
C9—Fe1—C4	108.1 (2)	C18—C19—Fe2	68.8 (3)
C6—Fe1—C4	152.7 (3)	C20—C19—H19	125.9
C18—Fe2—C17	40.9 (3)	C18—C19—H19	125.9
C18—Fe2—C12	115.9 (2)	Fe2—C19—H19	126.3
C17—Fe2—C12	105.6 (2)	C19—C20—C16	107.9 (6)
C18—Fe2—C16	67.9 (3)	C19—C20—Fe2	69.8 (3)
C17—Fe2—C16	40.8 (3)	C16—C20—Fe2	69.0 (3)
C12—Fe2—C16	128.1 (2)	C19—C20—H20	126
C18—Fe2—C11	150.5 (2)	C16—C20—H20	126
C17—Fe2—C11	117.1 (2)	Fe2—C20—H20	126.7
C12—Fe2—C11	41.20 (18)	C25—C21—C22	108.3 (5)
C16—Fe2—C11	109.1 (2)	C25—C21—Ti1	73.4 (3)
C18—Fe2—C15	165.0 (3)	C22—C21—Ti1	73.8 (3)
C17—Fe2—C15	153.7 (3)	C25—C21—H21	125.9
C12—Fe2—C15	69.16 (19)	C22—C21—H21	125.9
C16—Fe2—C15	121.2 (3)	Ti1—C21—H21	118.8
C11—Fe2—C15	41.91 (18)	C21—C22—C23	107.0 (5)
C18—Fe2—C19	40.2 (2)	C21—C22—Ti1	72.0 (3)
C17—Fe2—C19	68.6 (2)	C23—C22—Ti1	72.2 (3)
C12—Fe2—C19	149.7 (2)	C21—C22—H22	126.5
C16—Fe2—C19	67.8 (2)	C23—C22—H22	126.5
C11—Fe2—C19	168.2 (2)	Ti1—C22—H22	121.1
C15—Fe2—C19	129.0 (2)	C24—C23—C22	107.7 (5)
C18—Fe2—C20	67.4 (3)	C24—C23—Ti1	72.3 (3)
C17—Fe2—C20	68.7 (3)	C22—C23—Ti1	73.3 (3)
C12—Fe2—C20	167.8 (2)	C24—C23—H23	126.1
C16—Fe2—C20	40.7 (3)	C22—C23—H23	126.1
C11—Fe2—C20	130.7 (2)	Ti1—C23—H23	120.2
C15—Fe2—C20	110.9 (2)	C25—C24—C23	108.2 (5)
C19—Fe2—C20	39.7 (2)	C25—C24—Ti1	73.7 (3)
C18—Fe2—C13	105.7 (2)	C23—C24—Ti1	73.4 (3)
C17—Fe2—C13	125.3 (3)	C25—C24—H24	125.9
C12—Fe2—C13	40.68 (19)	C23—C24—H24	125.9
C16—Fe2—C13	164.6 (3)	Ti1—C24—H24	118.8
C11—Fe2—C13	69.11 (19)	C24—C25—C21	108.8 (5)
C15—Fe2—C13	68.3 (2)	C24—C25—Ti1	72.4 (3)
C19—Fe2—C13	117.0 (2)	C21—C25—Ti1	72.6 (3)
C20—Fe2—C13	151.4 (2)	C24—C25—H25	125.6
C18—Fe2—C14	126.7 (3)	C21—C25—H25	125.6
C17—Fe2—C14	163.9 (3)	Ti1—C25—H25	121.1
C12—Fe2—C14	68.6 (2)	C27—C26—C30	107.9 (5)
C16—Fe2—C14	154.2 (3)	C27—C26—Ti1	72.8 (3)
C11—Fe2—C14	69.13 (18)	C30—C26—Ti1	72.9 (3)
C15—Fe2—C14	40.08 (18)	C27—C26—H26	126
C19—Fe2—C14	108.4 (2)	C30—C26—H26	126
C20—Fe2—C14	119.9 (2)	Ti1—C26—H26	120.1
C13—Fe2—C14	40.7 (2)	C28—C27—C26	108.3 (5)
O2—Ti1—O1	92.65 (13)	C28—C27—Ti1	72.2 (3)
O2—Ti1—C29	104.37 (18)	C26—C27—Ti1	73.4 (3)
O1—Ti1—C29	137.61 (17)	C28—C27—H27	125.8
O2—Ti1—C28	133.11 (18)	C26—C27—H27	125.8
O1—Ti1—C28	108.47 (17)	Ti1—C27—H27	120.3
C29—Ti1—C28	34.52 (19)	C27—C28—C29	108.3 (5)
O2—Ti1—C24	108.04 (17)	C27—C28—Ti1	73.8 (3)
O1—Ti1—C24	133.64 (18)	C29—C28—Ti1	72.5 (3)
C29—Ti1—C24	77.5 (2)	C27—C28—H28	125.9
C28—Ti1—C24	87.2 (2)	C29—C28—H28	125.9
O2—Ti1—C21	87.78 (16)	Ti1—C28—H28	119.7
O1—Ti1—C21	84.05 (17)	C30—C29—C28	107.8 (5)
C29—Ti1—C21	134.2 (2)	C30—C29—Ti1	74.3 (3)
C28—Ti1—C21	134.48 (19)	C28—C29—Ti1	73.0 (3)
C24—Ti1—C21	56.81 (19)	C30—C29—H29	126.1
O2—Ti1—C23	137.34 (17)	C28—C29—H29	126.1
O1—Ti1—C23	105.30 (17)	Ti1—C29—H29	118.5
C29—Ti1—C23	88.1 (2)	C29—C30—C26	107.6 (5)
C28—Ti1—C23	77.5 (2)	C29—C30—Ti1	71.5 (3)
C24—Ti1—C23	34.30 (18)	C26—C30—Ti1	72.8 (3)
C21—Ti1—C23	56.99 (18)	C29—C30—H30	126.2
O2—Ti1—C25	80.88 (15)	C26—C30—H30	126.2
O1—Ti1—C25	117.48 (16)	Ti1—C30—H30	121.3
C29—Ti1—C25	103.6 (2)	C31—O3—C34	104.7 (6)
C28—Ti1—C25	120.79 (19)	C32—C31—O3	107.5 (7)
C24—Ti1—C25	33.85 (17)	C32—C31—H31A	110.2
C21—Ti1—C25	34.00 (18)	O3—C31—H31A	110.2
C23—Ti1—C25	56.47 (18)	C32—C31—H31B	110.2
O2—Ti1—C27	117.61 (16)	O3—C31—H31B	110.2
O1—Ti1—C27	80.76 (16)	H31A—C31—H31B	108.5
C29—Ti1—C27	56.91 (19)	C31—C32—C33	107.4 (7)
C28—Ti1—C27	33.97 (17)	C31—C32—H32A	110.2
C24—Ti1—C27	120.86 (19)	C33—C32—H32A	110.2
C21—Ti1—C27	150.79 (19)	C31—C32—H32B	110.2
C23—Ti1—C27	103.50 (19)	C33—C32—H32B	110.2
C25—Ti1—C27	154.67 (17)	H32A—C32—H32B	108.5
O2—Ti1—C26	84.27 (16)	C34—C33—C32	100.8 (7)
O1—Ti1—C26	87.39 (16)	C34—C33—H33A	111.6
C29—Ti1—C26	56.93 (19)	C32—C33—H33A	111.6
C28—Ti1—C26	56.53 (19)	C34—C33—H33B	111.6
C24—Ti1—C26	134.4 (2)	C32—C33—H33B	111.6
C21—Ti1—C26	168.03 (18)	H33A—C33—H33B	109.4
C23—Ti1—C26	133.89 (19)	C33—C34—O3	111.8 (6)
C25—Ti1—C26	151.40 (19)	C33—C34—H34A	109.3
C27—Ti1—C26	33.87 (17)	O3—C34—H34A	109.3
O2—Ti1—C30	76.41 (17)	C33—C34—H34B	109.3
O1—Ti1—C30	120.65 (18)	O3—C34—H34B	109.3
C29—Ti1—C30	34.20 (19)	H34A—C34—H34B	107.9
C28—Ti1—C30	56.7 (2)	C38—O4—C35	104.0 (7)
C24—Ti1—C30	104.6 (2)	C36—C35—O4	113.4 (8)
C21—Ti1—C30	150.7 (2)	C36—C35—H35A	108.9
C23—Ti1—C30	122.1 (2)	O4—C35—H35A	108.9
C25—Ti1—C30	117.8 (2)	C36—C35—H35B	108.9
C27—Ti1—C30	56.55 (19)	O4—C35—H35B	108.9
C26—Ti1—C30	34.21 (19)	H35A—C35—H35B	107.7
O2—Ti1—C22	121.15 (16)	C35—C36—C37	102.8 (7)
O1—Ti1—C22	76.61 (16)	C35—C36—H36A	111.2
C29—Ti1—C22	122.40 (19)	C37—C36—H36A	111.2
C28—Ti1—C22	104.56 (19)	C35—C36—H36B	111.2
C24—Ti1—C22	57.10 (19)	C37—C36—H36B	111.2
C21—Ti1—C22	34.23 (17)	H36A—C36—H36B	109.1
C23—Ti1—C22	34.54 (17)	C38—C37—C36	103.6 (7)
C25—Ti1—C22	56.56 (18)	C38—C37—H37A	111
C27—Ti1—C22	117.25 (18)	C36—C37—H37A	111
C26—Ti1—C22	150.02 (19)	C38—C37—H37B	111
C30—Ti1—C22	156.58 (19)	C36—C37—H37B	111
S2—S1—P1	102.78 (7)	H37A—C37—H37B	109
S1—S2—P2	103.87 (7)	O4—C38—C37	108.2 (8)
O1—P1—C1	105.0 (2)	O4—C38—H38A	110.1
O1—P1—S3	120.29 (14)	C37—C38—H38A	110.1
C1—P1—S3	113.95 (17)	O4—C38—H38B	110.1
O1—P1—S1	108.03 (13)	C37—C38—H38B	110.1
C1—P1—S1	106.09 (15)	H38A—C38—H38B	108.4
S3—P1—S1	102.57 (8)	C35F—O4F—C38F	99.6 (12)
O2—P2—C11	104.86 (19)	C36F—C35F—O4F	108.4 (11)
O2—P2—S4	120.75 (14)	C36F—C35F—H35C	110
C11—P2—S4	113.77 (17)	O4F—C35F—H35C	110
O2—P2—S2	106.70 (13)	C36F—C35F—H35D	110
C11—P2—S2	107.42 (16)	O4F—C35F—H35D	110
S4—P2—S2	102.50 (7)	H35C—C35F—H35D	108.4
P1—O1—Ti1	149.4 (2)	C35F—C36F—C37F	105.6 (12)
P2—O2—Ti1	151.0 (2)	C35F—C36F—H36C	110.6
C5—C1—C2	108.0 (4)	C37F—C36F—H36C	110.6
C5—C1—P1	127.4 (4)	C35F—C36F—H36D	110.6
C2—C1—P1	124.2 (4)	C37F—C36F—H36D	110.6
C5—C1—Fe1	69.9 (3)	H36C—C36F—H36D	108.7
C2—C1—Fe1	69.7 (3)	C38F—C37F—C36F	94.0 (12)
P1—C1—Fe1	120.4 (2)	C38F—C37F—H37C	112.9
C3—C2—C1	106.5 (4)	C36F—C37F—H37C	112.9
C3—C2—Fe1	69.8 (3)	C38F—C37F—H37D	112.9
C1—C2—Fe1	68.9 (3)	C36F—C37F—H37D	112.9
C3—C2—H2	126.7	H37C—C37F—H37D	110.3
C1—C2—H2	126.7	C37F—C38F—O4F	105.5 (13)
Fe1—C2—H2	126.2	C37F—C38F—H38C	110.6
C4—C3—C2	109.5 (4)	O4F—C38F—H38C	110.6
C4—C3—Fe1	70.5 (3)	C37F—C38F—H38D	110.6
C2—C3—Fe1	69.5 (3)	O4F—C38F—H38D	110.6
C4—C3—H3	125.3	H38C—C38F—H38D	108.8
C2—C3—H3	125.3	C39—O5—C42	100.3 (9)
Fe1—C3—H3	126.3	C40—C39—O5	113.7 (12)
C3—C4—C5	107.8 (4)	C40—C39—H39A	108.8
C3—C4—Fe1	69.4 (3)	O5—C39—H39A	108.8
C5—C4—Fe1	69.0 (3)	C40—C39—H39B	108.8
C3—C4—H4	126.1	O5—C39—H39B	108.8
C5—C4—H4	126.1	H39A—C39—H39B	107.7
Fe1—C4—H4	127.1	C39—C40—C41	103.3 (12)
C4—C5—C1	108.2 (4)	C39—C40—H40A	111.1
C4—C5—Fe1	70.5 (3)	C41—C40—H40A	111.1
C1—C5—Fe1	69.1 (3)	C39—C40—H40B	111.1
C4—C5—H5	125.9	C41—C40—H40B	111.1
C1—C5—H5	125.9	H40A—C40—H40B	109.1
Fe1—C5—H5	126	C42—C41—C40	103.7 (11)
C10—C6—C7	108.0 (6)	C42—C41—H41A	111
C10—C6—Fe1	69.8 (3)	C40—C41—H41A	111
C7—C6—Fe1	69.6 (3)	C42—C41—H41B	111
C10—C6—H6	126	C40—C41—H41B	111
C7—C6—H6	126	H41A—C41—H41B	109
Fe1—C6—H6	126.2	C41—C42—O5	109.9 (11)
C6—C7—C8	107.8 (6)	C41—C42—H42A	109.7
C6—C7—Fe1	70.3 (3)	O5—C42—H42A	109.7
C8—C7—Fe1	69.5 (3)	C41—C42—H42B	109.7
C6—C7—H7	126.1	O5—C42—H42B	109.7
C8—C7—H7	126.1	H42A—C42—H42B	108.2
Fe1—C7—H7	125.7	C43—O6—C46	96.8 (13)
C9—C8—C7	107.4 (6)	C44—C43—O6	105.6 (14)
C9—C8—Fe1	70.1 (3)	C44—C43—H43A	110.6
C7—C8—Fe1	69.3 (3)	O6—C43—H43A	110.6
C9—C8—H8	126.3	C44—C43—H43B	110.6
C7—C8—H8	126.3	O6—C43—H43B	110.6
Fe1—C8—H8	125.9	H43A—C43—H43B	108.8
C8—C9—C10	107.6 (5)	C43—C44—C45	103.2 (15)
C8—C9—Fe1	69.7 (3)	C43—C44—H44A	111.1
C10—C9—Fe1	69.6 (3)	C45—C44—H44A	111.1
C8—C9—H9	126.2	C43—C44—H44B	111.1
C10—C9—H9	126.2	C45—C44—H44B	111.1
Fe1—C9—H9	126	H44A—C44—H44B	109.1
C6—C10—C9	109.2 (6)	C46—C45—C44	83.9 (14)
C6—C10—Fe1	70.4 (3)	C46—C45—H45A	114.7
C9—C10—Fe1	70.1 (3)	C44—C45—H45A	114.7
C6—C10—H10	125.4	C46—C45—H45B	114.7
C9—C10—H10	125.4	C44—C45—H45B	114.7
Fe1—C10—H10	125.7	H45A—C45—H45B	111.7
C12—C11—C15	106.2 (4)	C45—C46—O6	98.7 (14)
C12—C11—P2	126.4 (4)	C45—C46—H46A	112
C15—C11—P2	127.2 (3)	O6—C46—H46A	112
C12—C11—Fe2	69.2 (3)	C45—C46—H46B	112
C15—C11—Fe2	69.2 (2)	O6—C46—H46B	112
P2—C11—Fe2	122.1 (2)	H46A—C46—H46B	109.7
C13—C12—C11	109.0 (4)		
			
P1—S1—S2—P2	−93.64 (7)	C17—Fe2—C15—C11	−44.5 (7)
S2—S1—P1—O1	51.64 (15)	C12—Fe2—C15—C11	38.6 (3)
S2—S1—P1—C1	−60.53 (18)	C16—Fe2—C15—C11	−84.1 (3)
S2—S1—P1—S3	179.66 (7)	C19—Fe2—C15—C11	−169.7 (3)
S1—S2—P2—O2	46.17 (15)	C20—Fe2—C15—C11	−128.3 (3)
S1—S2—P2—C11	−65.84 (17)	C13—Fe2—C15—C11	82.4 (3)
S1—S2—P2—S4	174.00 (7)	C14—Fe2—C15—C11	119.7 (4)
C1—P1—O1—Ti1	155.0 (4)	C18—Fe2—C16—C17	−38.9 (4)
S3—P1—O1—Ti1	−74.9 (4)	C12—Fe2—C16—C17	67.4 (5)
S1—P1—O1—Ti1	42.2 (4)	C11—Fe2—C16—C17	109.6 (4)
O2—Ti1—O1—P1	−50.6 (4)	C15—Fe2—C16—C17	154.3 (4)
C29—Ti1—O1—P1	64.1 (5)	C19—Fe2—C16—C17	−82.5 (4)
C28—Ti1—O1—P1	86.8 (4)	C20—Fe2—C16—C17	−119.4 (6)
C24—Ti1—O1—P1	−168.8 (3)	C13—Fe2—C16—C17	29.0 (11)
C21—Ti1—O1—P1	−138.1 (4)	C14—Fe2—C16—C17	−168.9 (5)
C23—Ti1—O1—P1	168.5 (4)	C18—Fe2—C16—C20	80.5 (4)
C25—Ti1—O1—P1	−131.7 (4)	C17—Fe2—C16—C20	119.4 (6)
C27—Ti1—O1—P1	66.9 (4)	C12—Fe2—C16—C20	−173.2 (3)
C26—Ti1—O1—P1	33.5 (4)	C11—Fe2—C16—C20	−131.0 (4)
C30—Ti1—O1—P1	25.1 (4)	C15—Fe2—C16—C20	−86.3 (4)
C22—Ti1—O1—P1	−172.1 (4)	C19—Fe2—C16—C20	36.9 (4)
C11—P2—O2—Ti1	163.5 (4)	C13—Fe2—C16—C20	148.4 (8)
S4—P2—O2—Ti1	−66.5 (4)	C14—Fe2—C16—C20	−49.5 (7)
S2—P2—O2—Ti1	49.7 (4)	C20—C16—C17—C18	0.0 (7)
O1—Ti1—O2—P2	−52.6 (4)	Fe2—C16—C17—C18	59.8 (4)
C29—Ti1—O2—P2	166.6 (4)	C20—C16—C17—Fe2	−59.7 (4)
C28—Ti1—O2—P2	−171.1 (4)	C12—Fe2—C17—C18	111.7 (4)
C24—Ti1—O2—P2	85.3 (4)	C16—Fe2—C17—C18	−117.2 (6)
C21—Ti1—O2—P2	31.3 (4)	C11—Fe2—C17—C18	154.4 (4)
C23—Ti1—O2—P2	63.5 (5)	C15—Fe2—C17—C18	−173.9 (4)
C25—Ti1—O2—P2	64.8 (4)	C19—Fe2—C17—C18	−36.8 (4)
C27—Ti1—O2—P2	−133.6 (4)	C20—Fe2—C17—C18	−79.6 (4)
C26—Ti1—O2—P2	−139.7 (4)	C13—Fe2—C17—C18	71.8 (5)
C30—Ti1—O2—P2	−173.5 (5)	C14—Fe2—C17—C18	45.3 (10)
C22—Ti1—O2—P2	23.3 (5)	C18—Fe2—C17—C16	117.2 (6)
O1—P1—C1—C5	−12.9 (5)	C12—Fe2—C17—C16	−131.0 (4)
S3—P1—C1—C5	−146.5 (4)	C11—Fe2—C17—C16	−88.3 (4)
S1—P1—C1—C5	101.4 (4)	C15—Fe2—C17—C16	−56.6 (7)
O1—P1—C1—C2	159.1 (4)	C19—Fe2—C17—C16	80.4 (4)
S3—P1—C1—C2	25.5 (4)	C20—Fe2—C17—C16	37.6 (4)
S1—P1—C1—C2	−86.6 (4)	C13—Fe2—C17—C16	−170.9 (3)
O1—P1—C1—Fe1	74.1 (3)	C14—Fe2—C17—C16	162.5 (7)
S3—P1—C1—Fe1	−59.6 (3)	C16—C17—C18—C19	−0.1 (7)
S1—P1—C1—Fe1	−171.6 (2)	Fe2—C17—C18—C19	60.2 (4)
C2—Fe1—C1—C5	−119.1 (4)	C16—C17—C18—Fe2	−60.3 (4)
C7—Fe1—C1—C5	156.4 (3)	C17—Fe2—C18—C19	−120.1 (6)
C10—Fe1—C1—C5	73.6 (4)	C12—Fe2—C18—C19	155.8 (3)
C3—Fe1—C1—C5	−80.7 (3)	C16—Fe2—C18—C19	−81.3 (4)
C8—Fe1—C1—C5	−167.0 (5)	C11—Fe2—C18—C19	−171.3 (4)
C9—Fe1—C1—C5	39.1 (10)	C15—Fe2—C18—C19	49.3 (10)
C6—Fe1—C1—C5	113.5 (3)	C20—Fe2—C18—C19	−37.0 (3)
C4—Fe1—C1—C5	−37.5 (3)	C13—Fe2—C18—C19	113.5 (4)
C5—Fe1—C1—C2	119.1 (4)	C14—Fe2—C18—C19	74.2 (4)
C7—Fe1—C1—C2	−84.5 (4)	C12—Fe2—C18—C17	−84.0 (5)
C10—Fe1—C1—C2	−167.3 (3)	C16—Fe2—C18—C17	38.8 (4)
C3—Fe1—C1—C2	38.3 (3)	C11—Fe2—C18—C17	−51.2 (7)
C8—Fe1—C1—C2	−47.9 (6)	C15—Fe2—C18—C17	169.4 (8)
C9—Fe1—C1—C2	158.2 (9)	C19—Fe2—C18—C17	120.1 (6)
C6—Fe1—C1—C2	−127.4 (3)	C20—Fe2—C18—C17	83.1 (5)
C4—Fe1—C1—C2	81.6 (3)	C13—Fe2—C18—C17	−126.4 (4)
C5—Fe1—C1—P1	−122.4 (4)	C14—Fe2—C18—C17	−165.7 (4)
C2—Fe1—C1—P1	118.6 (4)	C17—C18—C19—C20	0.1 (6)
C7—Fe1—C1—P1	34.0 (4)	Fe2—C18—C19—C20	59.7 (4)
C10—Fe1—C1—P1	−48.8 (4)	C17—C18—C19—Fe2	−59.7 (4)
C3—Fe1—C1—P1	156.9 (3)	C18—Fe2—C19—C20	−119.5 (5)
C8—Fe1—C1—P1	70.6 (6)	C17—Fe2—C19—C20	−82.0 (4)
C9—Fe1—C1—P1	−83.2 (10)	C12—Fe2—C19—C20	−166.3 (4)
C6—Fe1—C1—P1	−8.9 (4)	C16—Fe2—C19—C20	−37.9 (4)
C4—Fe1—C1—P1	−159.8 (4)	C11—Fe2—C19—C20	39.1 (11)
C5—C1—C2—C3	−0.2 (5)	C15—Fe2—C19—C20	75.1 (4)
P1—C1—C2—C3	−173.6 (3)	C13—Fe2—C19—C20	158.2 (3)
Fe1—C1—C2—C3	−59.9 (3)	C14—Fe2—C19—C20	114.9 (4)
C5—C1—C2—Fe1	59.7 (3)	C17—Fe2—C19—C18	37.5 (4)
P1—C1—C2—Fe1	−113.6 (3)	C12—Fe2—C19—C18	−46.8 (6)
C1—Fe1—C2—C3	117.9 (4)	C16—Fe2—C19—C18	81.6 (5)
C5—Fe1—C2—C3	80.1 (3)	C11—Fe2—C19—C18	158.6 (9)
C7—Fe1—C2—C3	−129.0 (4)	C15—Fe2—C19—C18	−165.4 (4)
C10—Fe1—C2—C3	168.1 (10)	C20—Fe2—C19—C18	119.5 (5)
C8—Fe1—C2—C3	−85.5 (4)	C13—Fe2—C19—C18	−82.3 (4)
C9—Fe1—C2—C3	−52.8 (6)	C14—Fe2—C19—C18	−125.6 (4)
C6—Fe1—C2—C3	−167.9 (4)	C18—C19—C20—C16	0.0 (6)
C4—Fe1—C2—C3	36.5 (3)	Fe2—C19—C20—C16	58.7 (4)
C5—Fe1—C2—C1	−37.8 (3)	C18—C19—C20—Fe2	−58.7 (4)
C7—Fe1—C2—C1	113.1 (3)	C17—C16—C20—C19	0.0 (7)
C10—Fe1—C2—C1	50.2 (11)	Fe2—C16—C20—C19	−59.2 (4)
C3—Fe1—C2—C1	−117.9 (4)	C17—C16—C20—Fe2	59.2 (4)
C8—Fe1—C2—C1	156.7 (3)	C18—Fe2—C20—C19	37.5 (4)
C9—Fe1—C2—C1	−170.7 (4)	C17—Fe2—C20—C19	81.8 (4)
C6—Fe1—C2—C1	74.2 (4)	C12—Fe2—C20—C19	145.6 (10)
C4—Fe1—C2—C1	−81.3 (3)	C16—Fe2—C20—C19	119.5 (6)
C1—C2—C3—C4	0.0 (5)	C11—Fe2—C20—C19	−170.2 (3)
Fe1—C2—C3—C4	−59.3 (3)	C15—Fe2—C20—C19	−126.5 (3)
C1—C2—C3—Fe1	59.3 (3)	C13—Fe2—C20—C19	−43.6 (6)
C1—Fe1—C3—C4	81.7 (3)	C14—Fe2—C20—C19	−83.0 (4)
C5—Fe1—C3—C4	37.5 (3)	C18—Fe2—C20—C16	−82.0 (5)
C2—Fe1—C3—C4	120.6 (4)	C17—Fe2—C20—C16	−37.7 (4)
C7—Fe1—C3—C4	−168.7 (3)	C12—Fe2—C20—C16	26.1 (13)
C10—Fe1—C3—C4	−53.9 (6)	C11—Fe2—C20—C16	70.3 (5)
C8—Fe1—C3—C4	−128.1 (3)	C15—Fe2—C20—C16	114.0 (5)
C9—Fe1—C3—C4	−85.9 (3)	C19—Fe2—C20—C16	−119.5 (6)
C6—Fe1—C3—C4	164.9 (9)	C13—Fe2—C20—C16	−163.1 (5)
C1—Fe1—C3—C2	−38.9 (3)	C14—Fe2—C20—C16	157.6 (4)
C5—Fe1—C3—C2	−83.1 (3)	O2—Ti1—C21—C25	76.9 (3)
C7—Fe1—C3—C2	70.7 (4)	O1—Ti1—C21—C25	169.8 (3)
C10—Fe1—C3—C2	−174.5 (4)	C29—Ti1—C21—C25	−31.1 (4)
C8—Fe1—C3—C2	111.2 (4)	C28—Ti1—C21—C25	−80.1 (4)
C9—Fe1—C3—C2	153.4 (3)	C24—Ti1—C21—C25	−36.4 (3)
C6—Fe1—C3—C2	44.3 (11)	C23—Ti1—C21—C25	−77.6 (3)
C4—Fe1—C3—C2	−120.6 (4)	C27—Ti1—C21—C25	−131.3 (4)
C2—C3—C4—C5	0.2 (5)	C26—Ti1—C21—C25	125.3 (9)
Fe1—C3—C4—C5	−58.5 (3)	C30—Ti1—C21—C25	20.3 (5)
C2—C3—C4—Fe1	58.7 (3)	C22—Ti1—C21—C25	−115.3 (4)
C1—Fe1—C4—C3	−81.7 (3)	O2—Ti1—C21—C22	−167.8 (3)
C5—Fe1—C4—C3	−119.6 (4)	O1—Ti1—C21—C22	−74.9 (3)
C2—Fe1—C4—C3	−37.1 (3)	C29—Ti1—C21—C22	84.2 (4)
C7—Fe1—C4—C3	39.0 (10)	C28—Ti1—C21—C22	35.2 (4)
C10—Fe1—C4—C3	153.8 (3)	C24—Ti1—C21—C22	78.9 (3)
C8—Fe1—C4—C3	70.8 (4)	C23—Ti1—C21—C22	37.7 (3)
C9—Fe1—C4—C3	111.2 (3)	C25—Ti1—C21—C22	115.3 (4)
C6—Fe1—C4—C3	−172.4 (4)	C27—Ti1—C21—C22	−16.0 (5)
C1—Fe1—C4—C5	37.9 (3)	C26—Ti1—C21—C22	−119.5 (9)
C2—Fe1—C4—C5	82.5 (3)	C30—Ti1—C21—C22	135.6 (4)
C7—Fe1—C4—C5	158.6 (9)	C25—C21—C22—C23	1.7 (6)
C10—Fe1—C4—C5	−86.7 (3)	Ti1—C21—C22—C23	−64.2 (3)
C3—Fe1—C4—C5	119.6 (4)	C25—C21—C22—Ti1	65.8 (4)
C8—Fe1—C4—C5	−169.6 (3)	O2—Ti1—C22—C21	14.3 (4)
C9—Fe1—C4—C5	−129.2 (3)	O1—Ti1—C22—C21	99.2 (3)
C6—Fe1—C4—C5	−52.8 (6)	C29—Ti1—C22—C21	−122.3 (3)
C3—C4—C5—C1	−0.4 (5)	C28—Ti1—C22—C21	−154.8 (3)
Fe1—C4—C5—C1	−59.1 (3)	C24—Ti1—C22—C21	−78.0 (3)
C3—C4—C5—Fe1	58.7 (3)	C23—Ti1—C22—C21	−115.3 (5)
C2—C1—C5—C4	0.4 (5)	C25—Ti1—C22—C21	−37.3 (3)
P1—C1—C5—C4	173.4 (3)	C27—Ti1—C22—C21	171.3 (3)
Fe1—C1—C5—C4	59.9 (3)	C26—Ti1—C22—C21	158.8 (4)
C2—C1—C5—Fe1	−59.6 (3)	C30—Ti1—C22—C21	−120.5 (5)
P1—C1—C5—Fe1	113.5 (4)	O2—Ti1—C22—C23	129.6 (3)
C1—Fe1—C5—C4	−119.3 (4)	O1—Ti1—C22—C23	−145.5 (3)
C2—Fe1—C5—C4	−81.1 (3)	C29—Ti1—C22—C23	−7.0 (4)
C7—Fe1—C5—C4	−168.9 (5)	C28—Ti1—C22—C23	−39.5 (4)
C10—Fe1—C5—C4	112.3 (3)	C24—Ti1—C22—C23	37.3 (3)
C3—Fe1—C5—C4	−37.2 (3)	C21—Ti1—C22—C23	115.3 (5)
C8—Fe1—C5—C4	35.2 (10)	C25—Ti1—C22—C23	78.0 (3)
C9—Fe1—C5—C4	70.9 (4)	C27—Ti1—C22—C23	−73.4 (4)
C6—Fe1—C5—C4	155.1 (3)	C26—Ti1—C22—C23	−85.9 (5)
C2—Fe1—C5—C1	38.2 (3)	C30—Ti1—C22—C23	−5.2 (7)
C7—Fe1—C5—C1	−49.6 (6)	C21—C22—C23—C24	−0.5 (6)
C10—Fe1—C5—C1	−128.3 (3)	Ti1—C22—C23—C24	−64.5 (4)
C3—Fe1—C5—C1	82.1 (3)	C21—C22—C23—Ti1	64.0 (3)
C8—Fe1—C5—C1	154.5 (9)	O2—Ti1—C23—C24	38.8 (4)
C9—Fe1—C5—C1	−169.8 (3)	O1—Ti1—C23—C24	150.3 (3)
C6—Fe1—C5—C1	−85.5 (3)	C29—Ti1—C23—C24	−70.5 (3)
C4—Fe1—C5—C1	119.3 (4)	C28—Ti1—C23—C24	−103.7 (4)
C1—Fe1—C6—C10	−129.6 (4)	C21—Ti1—C23—C24	78.1 (4)
C5—Fe1—C6—C10	−85.8 (4)	C25—Ti1—C23—C24	37.2 (3)
C2—Fe1—C6—C10	−172.0 (3)	C27—Ti1—C23—C24	−125.8 (3)
C7—Fe1—C6—C10	119.3 (5)	C26—Ti1—C23—C24	−108.3 (4)
C3—Fe1—C6—C10	152.2 (8)	C30—Ti1—C23—C24	−67.0 (4)
C8—Fe1—C6—C10	80.7 (4)	C22—Ti1—C23—C24	115.5 (5)
C9—Fe1—C6—C10	37.2 (4)	O2—Ti1—C23—C22	−76.7 (4)
C4—Fe1—C6—C10	−49.3 (7)	O1—Ti1—C23—C22	34.9 (3)
C1—Fe1—C6—C7	111.1 (4)	C29—Ti1—C23—C22	174.0 (4)
C5—Fe1—C6—C7	154.9 (3)	C28—Ti1—C23—C22	140.9 (4)
C2—Fe1—C6—C7	68.7 (4)	C24—Ti1—C23—C22	−115.5 (5)
C10—Fe1—C6—C7	−119.3 (5)	C21—Ti1—C23—C22	−37.3 (3)
C3—Fe1—C6—C7	32.9 (11)	C25—Ti1—C23—C22	−78.3 (3)
C8—Fe1—C6—C7	−38.5 (4)	C27—Ti1—C23—C22	118.8 (3)
C9—Fe1—C6—C7	−82.0 (4)	C26—Ti1—C23—C22	136.2 (3)
C4—Fe1—C6—C7	−168.5 (4)	C30—Ti1—C23—C22	177.6 (3)
C10—C6—C7—C8	0.2 (6)	C22—C23—C24—C25	−0.9 (6)
Fe1—C6—C7—C8	59.6 (4)	Ti1—C23—C24—C25	−66.1 (4)
C10—C6—C7—Fe1	−59.4 (4)	C22—C23—C24—Ti1	65.2 (4)
C1—Fe1—C7—C6	−86.9 (4)	O2—Ti1—C24—C25	−38.3 (4)
C5—Fe1—C7—C6	−52.5 (6)	O1—Ti1—C24—C25	73.9 (4)
C2—Fe1—C7—C6	−130.5 (3)	C29—Ti1—C24—C25	−139.5 (4)
C10—Fe1—C7—C6	37.3 (3)	C28—Ti1—C24—C25	−173.0 (4)
C3—Fe1—C7—C6	−170.7 (3)	C21—Ti1—C24—C25	36.6 (3)
C8—Fe1—C7—C6	118.7 (5)	C23—Ti1—C24—C25	115.2 (5)
C9—Fe1—C7—C6	81.0 (4)	C27—Ti1—C24—C25	−178.0 (3)
C4—Fe1—C7—C6	158.2 (8)	C26—Ti1—C24—C25	−138.2 (3)
C1—Fe1—C7—C8	154.4 (4)	C30—Ti1—C24—C25	−118.5 (3)
C5—Fe1—C7—C8	−171.1 (4)	C22—Ti1—C24—C25	77.7 (4)
C2—Fe1—C7—C8	110.8 (4)	O2—Ti1—C24—C23	−153.5 (3)
C10—Fe1—C7—C8	−81.4 (4)	O1—Ti1—C24—C23	−41.3 (4)
C3—Fe1—C7—C8	70.6 (4)	C29—Ti1—C24—C23	105.2 (3)
C9—Fe1—C7—C8	−37.7 (4)	C28—Ti1—C24—C23	71.8 (4)
C6—Fe1—C7—C8	−118.7 (5)	C21—Ti1—C24—C23	−78.7 (4)
C4—Fe1—C7—C8	39.5 (11)	C25—Ti1—C24—C23	−115.2 (5)
C6—C7—C8—C9	−0.1 (6)	C27—Ti1—C24—C23	66.8 (4)
Fe1—C7—C8—C9	60.0 (4)	C26—Ti1—C24—C23	106.6 (4)
C6—C7—C8—Fe1	−60.2 (4)	C30—Ti1—C24—C23	126.3 (3)
C1—Fe1—C8—C9	−171.4 (4)	C22—Ti1—C24—C23	−37.6 (3)
C5—Fe1—C8—C9	44.8 (11)	C23—C24—C25—C21	1.9 (6)
C2—Fe1—C8—C9	155.3 (3)	Ti1—C24—C25—C21	−64.0 (4)
C7—Fe1—C8—C9	−118.4 (5)	C23—C24—C25—Ti1	65.9 (4)
C10—Fe1—C8—C9	−37.8 (3)	C22—C21—C25—C24	−2.2 (6)
C3—Fe1—C8—C9	112.4 (4)	Ti1—C21—C25—C24	63.8 (4)
C6—Fe1—C8—C9	−81.0 (4)	C22—C21—C25—Ti1	−66.1 (3)
C4—Fe1—C8—C9	72.9 (4)	O2—Ti1—C25—C24	143.4 (4)
C1—Fe1—C8—C7	−53.0 (7)	O1—Ti1—C25—C24	−128.4 (3)
C5—Fe1—C8—C7	163.3 (8)	C29—Ti1—C25—C24	40.7 (4)
C2—Fe1—C8—C7	−86.3 (4)	C28—Ti1—C25—C24	8.1 (4)
C10—Fe1—C8—C7	80.6 (4)	C21—Ti1—C25—C24	−117.0 (5)
C3—Fe1—C8—C7	−129.2 (4)	C23—Ti1—C25—C24	−37.7 (3)
C9—Fe1—C8—C7	118.4 (5)	C27—Ti1—C25—C24	4.1 (6)
C6—Fe1—C8—C7	37.4 (4)	C26—Ti1—C25—C24	83.8 (5)
C4—Fe1—C8—C7	−168.7 (4)	C30—Ti1—C25—C24	74.1 (4)
C7—C8—C9—C10	0.0 (6)	C22—Ti1—C25—C24	−79.4 (4)
Fe1—C8—C9—C10	59.6 (3)	O2—Ti1—C25—C21	−99.7 (3)
C7—C8—C9—Fe1	−59.5 (4)	O1—Ti1—C25—C21	−11.4 (3)
C1—Fe1—C9—C8	161.0 (9)	C29—Ti1—C25—C21	157.6 (3)
C5—Fe1—C9—C8	−167.0 (4)	C28—Ti1—C25—C21	125.1 (3)
C2—Fe1—C9—C8	−48.4 (6)	C24—Ti1—C25—C21	117.0 (5)
C7—Fe1—C9—C8	38.6 (4)	C23—Ti1—C25—C21	79.3 (3)
C10—Fe1—C9—C8	118.8 (5)	C27—Ti1—C25—C21	121.1 (4)
C3—Fe1—C9—C8	−84.3 (4)	C26—Ti1—C25—C21	−159.3 (4)
C6—Fe1—C9—C8	82.0 (4)	C30—Ti1—C25—C21	−168.9 (3)
C4—Fe1—C9—C8	−126.8 (4)	C22—Ti1—C25—C21	37.6 (3)
C1—Fe1—C9—C10	42.2 (11)	O2—Ti1—C26—C27	170.2 (3)
C5—Fe1—C9—C10	74.2 (4)	O1—Ti1—C26—C27	77.3 (3)
C2—Fe1—C9—C10	−167.2 (4)	C29—Ti1—C26—C27	−78.5 (3)
C7—Fe1—C9—C10	−80.1 (4)	C28—Ti1—C26—C27	−37.0 (3)
C3—Fe1—C9—C10	157.0 (3)	C24—Ti1—C26—C27	−80.1 (4)
C8—Fe1—C9—C10	−118.8 (5)	C21—Ti1—C26—C27	121.6 (9)
C6—Fe1—C9—C10	−36.8 (4)	C23—Ti1—C26—C27	−31.6 (4)
C4—Fe1—C9—C10	114.4 (4)	C25—Ti1—C26—C27	−130.9 (4)
C7—C6—C10—C9	−0.2 (6)	C30—Ti1—C26—C27	−115.6 (5)
Fe1—C6—C10—C9	−59.5 (3)	C22—Ti1—C26—C27	20.1 (5)
C7—C6—C10—Fe1	59.3 (4)	O2—Ti1—C26—C30	−74.2 (3)
C8—C9—C10—C6	0.1 (6)	O1—Ti1—C26—C30	−167.1 (3)
Fe1—C9—C10—C6	59.7 (4)	C29—Ti1—C26—C30	37.1 (3)
C8—C9—C10—Fe1	−59.6 (4)	C28—Ti1—C26—C30	78.7 (3)
C1—Fe1—C10—C6	71.0 (5)	C24—Ti1—C26—C30	35.5 (4)
C5—Fe1—C10—C6	112.9 (4)	C21—Ti1—C26—C30	−122.8 (9)
C2—Fe1—C10—C6	29.7 (13)	C23—Ti1—C26—C30	84.1 (4)
C7—Fe1—C10—C6	−37.5 (4)	C25—Ti1—C26—C30	−15.3 (5)
C3—Fe1—C10—C6	−166.8 (5)	C27—Ti1—C26—C30	115.6 (5)
C8—Fe1—C10—C6	−82.3 (4)	C22—Ti1—C26—C30	135.8 (4)
C9—Fe1—C10—C6	−120.0 (5)	C30—C26—C27—C28	−0.7 (6)
C4—Fe1—C10—C6	156.5 (4)	Ti1—C26—C27—C28	64.2 (4)
C1—Fe1—C10—C9	−169.0 (3)	C30—C26—C27—Ti1	−65.0 (3)
C5—Fe1—C10—C9	−127.1 (3)	O2—Ti1—C27—C28	−127.2 (3)
C2—Fe1—C10—C9	149.7 (10)	O1—Ti1—C27—C28	144.7 (3)
C7—Fe1—C10—C9	82.5 (4)	C29—Ti1—C27—C28	−37.5 (3)
C3—Fe1—C10—C9	−46.8 (6)	C24—Ti1—C27—C28	8.9 (4)
C8—Fe1—C10—C9	37.7 (4)	C21—Ti1—C27—C28	85.1 (5)
C6—Fe1—C10—C9	120.0 (5)	C23—Ti1—C27—C28	41.0 (4)
C4—Fe1—C10—C9	−83.5 (4)	C25—Ti1—C27—C28	6.2 (6)
O2—P2—C11—C12	154.8 (4)	C26—Ti1—C27—C28	−116.1 (5)
S4—P2—C11—C12	20.8 (5)	C30—Ti1—C27—C28	−78.7 (4)
S2—P2—C11—C12	−91.9 (4)	C22—Ti1—C27—C28	75.0 (4)
O2—P2—C11—C15	−18.9 (5)	O2—Ti1—C27—C26	−11.0 (4)
S4—P2—C11—C15	−152.9 (4)	O1—Ti1—C27—C26	−99.2 (3)
S2—P2—C11—C15	94.4 (4)	C29—Ti1—C27—C26	78.6 (4)
O2—P2—C11—Fe2	68.2 (3)	C28—Ti1—C27—C26	116.1 (5)
S4—P2—C11—Fe2	−65.8 (3)	C24—Ti1—C27—C26	125.0 (3)
S2—P2—C11—Fe2	−178.5 (2)	C21—Ti1—C27—C26	−158.8 (4)
C18—Fe2—C11—C12	−47.8 (6)	C23—Ti1—C27—C26	157.2 (3)
C17—Fe2—C11—C12	−82.8 (4)	C25—Ti1—C27—C26	122.3 (5)
C16—Fe2—C11—C12	−126.5 (4)	C30—Ti1—C27—C26	37.4 (3)
C15—Fe2—C11—C12	117.7 (4)	C22—Ti1—C27—C26	−168.8 (3)
C19—Fe2—C11—C12	160.8 (9)	C26—C27—C28—C29	−0.2 (6)
C20—Fe2—C11—C12	−167.1 (3)	Ti1—C27—C28—C29	64.8 (4)
C13—Fe2—C11—C12	37.2 (3)	C26—C27—C28—Ti1	−65.0 (3)
C14—Fe2—C11—C12	80.9 (3)	O2—Ti1—C28—C27	75.3 (4)
C18—Fe2—C11—C15	−165.4 (5)	O1—Ti1—C28—C27	−37.0 (4)
C17—Fe2—C11—C15	159.6 (4)	C29—Ti1—C28—C27	115.7 (5)
C12—Fe2—C11—C15	−117.7 (4)	C24—Ti1—C28—C27	−172.4 (3)
C16—Fe2—C11—C15	115.8 (4)	C21—Ti1—C28—C27	−137.0 (3)
C19—Fe2—C11—C15	43.1 (11)	C23—Ti1—C28—C27	−139.2 (4)
C20—Fe2—C11—C15	75.3 (4)	C25—Ti1—C28—C27	−176.9 (3)
C13—Fe2—C11—C15	−80.4 (3)	C26—Ti1—C28—C27	36.9 (3)
C14—Fe2—C11—C15	−36.8 (3)	C30—Ti1—C28—C27	78.1 (4)
C18—Fe2—C11—P2	73.0 (6)	C22—Ti1—C28—C27	−117.5 (3)
C17—Fe2—C11—P2	37.9 (4)	O2—Ti1—C28—C29	−40.4 (4)
C12—Fe2—C11—P2	120.7 (4)	O1—Ti1—C28—C29	−152.7 (3)
C16—Fe2—C11—P2	−5.8 (4)	C24—Ti1—C28—C29	71.9 (4)
C15—Fe2—C11—P2	−121.6 (4)	C21—Ti1—C28—C29	107.2 (4)
C19—Fe2—C11—P2	−78.5 (10)	C23—Ti1—C28—C29	105.1 (4)
C20—Fe2—C11—P2	−46.4 (4)	C25—Ti1—C28—C29	67.3 (4)
C13—Fe2—C11—P2	157.9 (3)	C27—Ti1—C28—C29	−115.7 (5)
C14—Fe2—C11—P2	−158.4 (3)	C26—Ti1—C28—C29	−78.9 (4)
C15—C11—C12—C13	−0.3 (5)	C30—Ti1—C28—C29	−37.6 (3)
P2—C11—C12—C13	−175.1 (3)	C22—Ti1—C28—C29	126.8 (3)
Fe2—C11—C12—C13	−59.9 (3)	C27—C28—C29—C30	1.1 (6)
C15—C11—C12—Fe2	59.6 (3)	Ti1—C28—C29—C30	66.8 (4)
P2—C11—C12—Fe2	−115.2 (4)	C27—C28—C29—Ti1	−65.7 (4)
C18—Fe2—C12—C13	−84.0 (4)	O2—Ti1—C29—C30	36.1 (4)
C17—Fe2—C12—C13	−126.6 (4)	O1—Ti1—C29—C30	−74.5 (4)
C16—Fe2—C12—C13	−165.4 (4)	C28—Ti1—C29—C30	−114.7 (5)
C11—Fe2—C12—C13	119.9 (4)	C24—Ti1—C29—C30	141.8 (4)
C15—Fe2—C12—C13	80.6 (3)	C21—Ti1—C29—C30	137.2 (4)
C19—Fe2—C12—C13	−52.5 (6)	C23—Ti1—C29—C30	174.7 (4)
C20—Fe2—C12—C13	173.3 (10)	C25—Ti1—C29—C30	119.9 (4)
C14—Fe2—C12—C13	37.5 (3)	C27—Ti1—C29—C30	−77.8 (4)
C18—Fe2—C12—C11	156.1 (3)	C26—Ti1—C29—C30	−37.1 (3)
C17—Fe2—C12—C11	113.5 (4)	C22—Ti1—C29—C30	178.7 (3)
C16—Fe2—C12—C11	74.7 (4)	O2—Ti1—C29—C28	150.8 (3)
C15—Fe2—C12—C11	−39.3 (3)	O1—Ti1—C29—C28	40.2 (5)
C19—Fe2—C12—C11	−172.4 (4)	C24—Ti1—C29—C28	−103.5 (4)
C20—Fe2—C12—C11	53.4 (11)	C21—Ti1—C29—C28	−108.1 (4)
C13—Fe2—C12—C11	−119.9 (4)	C23—Ti1—C29—C28	−70.6 (4)
C14—Fe2—C12—C11	−82.4 (3)	C25—Ti1—C29—C28	−125.4 (3)
C11—C12—C13—C14	−0.1 (5)	C27—Ti1—C29—C28	36.9 (3)
Fe2—C12—C13—C14	−59.4 (3)	C26—Ti1—C29—C28	77.6 (4)
C11—C12—C13—Fe2	59.2 (3)	C30—Ti1—C29—C28	114.7 (5)
C18—Fe2—C13—C12	111.7 (4)	C22—Ti1—C29—C28	−66.6 (4)
C17—Fe2—C13—C12	71.4 (4)	C28—C29—C30—C26	−1.5 (6)
C16—Fe2—C13—C12	48.6 (10)	Ti1—C29—C30—C26	64.3 (3)
C11—Fe2—C13—C12	−37.7 (3)	C28—C29—C30—Ti1	−65.9 (4)
C15—Fe2—C13—C12	−82.8 (3)	C27—C26—C30—C29	1.4 (6)
C19—Fe2—C13—C12	153.3 (3)	Ti1—C26—C30—C29	−63.4 (3)
C20—Fe2—C13—C12	−177.0 (4)	C27—C26—C30—Ti1	64.8 (3)
C14—Fe2—C13—C12	−119.6 (4)	O2—Ti1—C30—C29	−144.1 (4)
C18—Fe2—C13—C14	−128.7 (3)	O1—Ti1—C30—C29	131.0 (3)
C17—Fe2—C13—C14	−169.0 (3)	C28—Ti1—C30—C29	38.0 (3)
C12—Fe2—C13—C14	119.6 (4)	C24—Ti1—C30—C29	−38.6 (4)
C16—Fe2—C13—C14	168.1 (9)	C21—Ti1—C30—C29	−84.9 (5)
C11—Fe2—C13—C14	81.9 (3)	C23—Ti1—C30—C29	−6.2 (4)
C15—Fe2—C13—C14	36.8 (3)	C25—Ti1—C30—C29	−72.3 (4)
C19—Fe2—C13—C14	−87.1 (3)	C27—Ti1—C30—C29	78.9 (4)
C20—Fe2—C13—C14	−57.5 (5)	C26—Ti1—C30—C29	116.0 (5)
C12—C13—C14—C15	0.5 (5)	C22—Ti1—C30—C29	−2.7 (7)
Fe2—C13—C14—C15	−57.9 (3)	O2—Ti1—C30—C26	100.0 (3)
C12—C13—C14—Fe2	58.4 (3)	O1—Ti1—C30—C26	15.0 (4)
C18—Fe2—C14—C15	−170.3 (3)	C29—Ti1—C30—C26	−116.0 (5)
C17—Fe2—C14—C15	154.2 (8)	C28—Ti1—C30—C26	−78.0 (3)
C12—Fe2—C14—C15	82.7 (3)	C24—Ti1—C30—C26	−154.6 (3)
C16—Fe2—C14—C15	−52.6 (6)	C21—Ti1—C30—C26	159.1 (4)
C11—Fe2—C14—C15	38.4 (3)	C23—Ti1—C30—C26	−122.2 (3)
C19—Fe2—C14—C15	−129.4 (3)	C25—Ti1—C30—C26	171.8 (3)
C20—Fe2—C14—C15	−87.5 (3)	C27—Ti1—C30—C26	−37.0 (3)
C13—Fe2—C14—C15	120.2 (4)	C22—Ti1—C30—C26	−118.7 (5)
C18—Fe2—C14—C13	69.5 (4)	C34—O3—C31—C32	28.7 (11)
C17—Fe2—C14—C13	34.0 (9)	O3—C31—C32—C33	−25.5 (12)
C12—Fe2—C14—C13	−37.5 (3)	C31—C32—C33—C34	11.7 (10)
C16—Fe2—C14—C13	−172.8 (5)	C32—C33—C34—O3	6.1 (9)
C11—Fe2—C14—C13	−81.8 (3)	C31—O3—C34—C33	−21.5 (10)
C15—Fe2—C14—C13	−120.2 (4)	C38—O4—C35—C36	−22.7 (12)
C19—Fe2—C14—C13	110.3 (3)	O4—C35—C36—C37	7.3 (11)
C20—Fe2—C14—C13	152.3 (3)	C35—C36—C37—C38	10.6 (11)
C13—C14—C15—C11	−0.7 (5)	C35—O4—C38—C37	29.3 (11)
Fe2—C14—C15—C11	−58.9 (3)	C36—C37—C38—O4	−24.8 (11)
C13—C14—C15—Fe2	58.1 (3)	C38F—O4F—C35F—C36F	−22 (3)
C12—C11—C15—C14	0.6 (5)	O4F—C35F—C36F—C37F	−9 (3)
P2—C11—C15—C14	175.4 (4)	C35F—C36F—C37F—C38F	35 (3)
Fe2—C11—C15—C14	60.2 (3)	C36F—C37F—C38F—O4F	−50 (2)
C12—C11—C15—Fe2	−59.6 (3)	C35F—O4F—C38F—C37F	48 (2)
P2—C11—C15—Fe2	115.1 (4)	C42—O5—C39—C40	−20.0 (18)
C18—Fe2—C15—C14	31.6 (10)	O5—C39—C40—C41	31 (2)
C17—Fe2—C15—C14	−164.2 (6)	C39—C40—C41—C42	−28 (2)
C12—Fe2—C15—C14	−81.1 (3)	C40—C41—C42—O5	17 (2)
C16—Fe2—C15—C14	156.2 (3)	C39—O5—C42—C41	−0.3 (17)
C11—Fe2—C15—C14	−119.7 (4)	C46—O6—C43—C44	17 (3)
C19—Fe2—C15—C14	70.6 (4)	O6—C43—C44—C45	22 (3)
C20—Fe2—C15—C14	112.0 (3)	C43—C44—C45—C46	−56 (3)
C13—Fe2—C15—C14	−37.3 (3)	C44—C45—C46—O6	65.3 (16)
C18—Fe2—C15—C11	151.3 (8)	C43—O6—C46—C45	−60 (2)

## References

[bb1] Brandenburg, K. (2005). *DIAMOND* Crystal Impact GbR, Bonn, Germany.

[bb2] Farrugia, L. J. (1999). *J. Appl. Cryst.* **32**, 837–838.

[bb3] Flack, H. D. (1983). *Acta Cryst.* A**39**, 876–881.

[bb4] Oxford Diffraction (2009). *CrysAlis PRO* Oxford Diffraction Ltd, Yarnton, England.

[bb5] Sheldrick, G. M. (2008). *Acta Cryst.* A**64**, 112–122. 10.1107/S0108767307043930 18156677

[bb6] Zank, G. A. & Rauchfuss, T. (1984). *Organometallics*, **3**, 1191–1199.

[bb7] Zank, G. A. & Rauchfuss, T. (1985). *Inorg. Chem.* **25**, 1431–1435.

